# O04 A systematic review of the effect of therapeutic drug monitoring on patient health outcomes during treatment with penicillins

**DOI:** 10.1093/jacamr/dlac003.003

**Published:** 2022-02-16

**Authors:** Timothy Luxton, Natalie King, Christoph Wälti, Lars Jeuken, Jonathan Sandoe

**Affiliations:** School of Biomedical Sciences, University of Leeds, Leeds, UK; Leeds Institute of Health Sciences, University of Leeds, Leeds, UK; School of Electronic and Electrical Engineering, University of Leeds, Leeds, UK; School of Biomedical Sciences, University of Leeds, Leeds, UK; Leiden Institute of Chemistry, Leiden University, PO Box 9502, 2300 RA, Leiden, the Netherlands; School of Medicine, University of Leeds, Leeds, UK

## Abstract

**Background:**

Dosing regimens guided by therapeutic drug monitoring (TDM) may be able to improve penicillin exposure in patients. Improved penicillin exposure could result in improved patient health outcomes. This systematic review aims to describe the impact TDM in penicillin treatment has on health outcomes, including the emergence of antimicrobial resistance (AMR).

**Methods:**

A search of four databases was conducted and supplemented with a hand search of the literature. Studies that measured concentrations of penicillins in patients, adjusted dosing regimens according to the result and reported health outcomes were selected. The risk of bias was assessed according to study type: randomized controlled trials (RCTs) were assessed with the revised Cochrane risk-of-bias tool for randomized trials (RoB2) assessment tool, the risk of bias in non-randomized studies (ROBINS-1) assessment tool was used to assess observational studies, and the Office of Health Assessment and Translation (OHAT) tool was used to assess case studies. The study characteristics were tabulated and described using a narrative synthesis.

**Results:**

Three RCTs, 16 observational cohort studies and 9 case studies were included. None of the studies showed statistically significant improvements in health outcomes, when comparing groups receiving TDM and standard care. Five observational studies showed improvement in at least one health outcome statistically significantly associated with target attainment: mortality,^1–5^ bacterial persistence,^1,4,5^ ICU length of stay,^4^ treatment efficacy,^1,4,6^ and suppression of AMR.^4^ However there was a high risk of bias in all of these studies for health outcomes. Most had pharmacological primary outcomes and were underpowered to detect health outcomes. All three RCTs and four (25%) observational studies found that the use of penicillin TDM was associated with improved pharmacological target attainment. Only one study was found that assessed the impact of β-lactams TDM on AMR and found that target attainment of 100% ƒ*T*>_4×MIC_ was significantly associated with suppression of resistance. No studies found a detrimental effect of penicillin TDM on health outcomes. A meta-analysis was not performed due to the heterogeneity of the included studies.

**Conclusions:**

There is little evidence to suggest that TDM improves health outcomes, however neither health outcomes nor reductions in emergence of AMR were adequately addressed by currently published studies. The evaluation of health outcomes was not the primary aim of the majority of the included studies, consequently those that did include these outcomes were underpowered to detect a difference. Observational studies often did not take into account co-interventions, or confounding factors, resulting in a high risk of bias. Large variations in how TDM of penicillins was implemented and the different populations used meant that a quantitative synthesis of results was not suitable. Recommendations to standardize penicillin TDM are a challenge currently, as there is no clear evidence of optimal conditions. Suitably powered studies designed to assess clinical outcomes are required to resolve the ambiguity surrounding the impacts of TDM on clinical outcomes, and to address the gap of the impacts on AMR. Further, appropriate standardized protocols and concentration targets for penicillin TDM in humans need to be identified for it to be implemented successfully.

